# Serum anti-Müllerian hormone levels are associated with perinatal outcomes in women undergoing IVF/ICSI: A multicenter retrospective cohort study

**DOI:** 10.3389/fendo.2023.1081069

**Published:** 2023-02-21

**Authors:** Yi-Chen He, Kai-Zhen Su, Jie Cai, Qing-Xia Meng, Yan-Ting Wu, He-Feng Huang

**Affiliations:** ^1^ Obstetrics and Gynecology Hospital, Institute of Reproduction and Development, Fudan University, Shanghai, China; ^2^ International Peace Maternity and Child Health Hospital, School of Medicine, Shanghai Jiao Tong University, Shanghai, China; ^3^ Department of Reproductive Medicine, Ningbo Women and Children’s Hospital, Ningbo, China; ^4^ Center of Reproduction and Genetics, The Affiliated Suzhou Hospital of Nanjing Medical University, Suzhou Municipal Hospital, Suzhou, China; ^5^ Research Units of Embryo Original Diseases, Chinese Academy of Medical Sciences, Shanghai, China

**Keywords:** anti-Müllerian hormone, *in vitro* fertilization, intracytoplasmic sperm injection, perinatal outcomes, intrahepatic cholestasis of pregnancy, gestational diabetes mellitus, pregnancy-induced hypertension

## Abstract

**Introduction:**

Anti-Müllerian hormone (AMH) level has long been considered as a serum biomarker of ovarian reserve clinically, while emerging data suggest that serum AMH level may also predict pregnancy outcomes. However, whether pregestational serum AMH levels are related to perinatal outcomes among women undergoing *in vitro* fertilization (IVF)/intracytoplasmic sperm injection (ICSI) cycles is unknown.

**Objective:**

To explore the association between different AMH levels and perinatal outcomes in women with live births in IVF/ICSI.

**Methods:**

This multicenter retrospective cohort study was conducted among three different provinces in China, from January 2014 to October 2019. A total of 13,763 IVF/ICSI cycles with 5657 live-delivery pregnant women and 6797 newborns were recruited. Participants were categorized into three groups according to the <25th (low), 25 to 75th (average), and >75th (high) percentile of serum AMH concentration. Perinatal outcomes were compared among groups. Subgroup analyses were conducted based on the number of live births.

**Results:**

Among women with singleton deliveries, low and high AMH levels increased the risk of intrahepatic cholestasis of pregnancy (ICP) (aOR1 = 6.02, 95%CI: 2.10-17.22; aOR2 = 3.65, 95%CI:1.32-10.08) and decreased the risk of macrosomia (aOR1 = 0.65, 95%CI:0.48-0.89; aOR2 = 0.72, 95%CI:0.57-0.96), while low AMH reduced the risk of large for gestational age (LGA, aOR=0.74, 95%CI:0.59-0.93) and premature rupture of membrane (PROM, aOR=0.50, 95%CI:0.31-0.79)compared with the average AMH group. In women with multiple deliveries, high AMH levels increased the risks of gestational diabetes mellitus (GDM, aOR=2.40, 95%CI:1.48-3.91) and pregnancy-induced hypertension (PIH, aOR=2.26, 95%CI:1.20-4.22) compared with the average AMH group, while low AMH levels increased the risk of ICP (aOR=14.83, 95%CI:1.92-54.30). However, there was no evidence of differences in preterm birth, congenital anomaly, and other perinatal outcomes among the three groups in both singleton and multiple deliveries.

**Conclusions:**

Abnormal AMH levels increased the risk of ICP regardless of the number of live births for women undergoing IVF/ICSI, while high AMH levels increased the risks of GDM and PIH in multiple deliveries. However, serum AMH levels were not associated with adverse neonatal outcomes in IVF/ICSI. The underlying mechanism warrants further investigation.

## Introduction

Anti-Müllerian hormone (AMH), mostly secreted by granulosa cells of preantral and early antral follicles, is a dimeric glycoprotein belonging to the family of transforming growth factor beta (TGF-β) (1, 2). During follicular development, AMH can inhibit the recruitment of initial follicles as well as participate in the regulation of follicular selection (3, 4). Lines of evidence demonstrated serum AMH is linearly related to the number of developing follicles as well as remaining relatively stable during the menstrual cycle. Thus, AMH is widely used as a serum marker of ovarian reserve *in vitro* fertilization (IVF) (1, 5, 6). However, the relationship of AMH to the quality of the oocyte pool and pregnancy outcomes remains unclear (7).

The interest in the impact of serum AMH levels on pregnancy outcomes has emerged in the last few years. Despite several retrospective cohorts pointing to serum AMH as a weak predictor of live birth after assisted reproductive technology (ART) (low AMH level is associated with decreased live birth), only a few studies focus on pregnancy complications and neonatal outcomes (8, 9). A cohort study based on the serum AMH collected in the first trimester has demonstrated that low maternal level of AMH is a predictor of pregnancy-induced hypertension (PIH) in naturally conceived women, while associations in other complications included gestational diabetes (GDM), preterm birth and small for gestational age (SGA) were not identified (10). It is interesting to note that recent studies have reported a significant association between AMH and preterm delivery in patients with polycystic ovarian syndrome (PCOS) after IVF (11, 12), suggesting its potential to be a marker of preterm delivery.

Considering the discrepancies and limited sample size, we want to elucidate if AMH is related to pregnancy outcomes, especially in women conceived with ART. ART has been increasingly used for infertile couples thanks to the advances in technology and provision of services, resulting in more than 300 thousand infants born through it each year in China (13). While ART affords patients the opportunity to have biologically-related children, potential risks including GDM, PIH, preterm birth and low-birth-weight (LBW) exist as results of the laboratory procedures and genetic background (14–18). Given the general use of AMH to assess ovarian reserve before ART, we hope the test will be given new insights as a marker of perinatal outcomes in specific aspects.

To further analyze the effect of AMH on adverse perinatal outcomes among ART pregnancies, we conducted a multi-center retrospective cohort study of women who underwent IVF/intracytoplasmic sperm injection (ICSI) cycles in different AMH groups.

## Methods

### Study design and participants

This retrospective, multi-center cohort study was conducted on women who underwent IVF/ICSI cycles and achieved live births from January 2014 to October 2019 in three study centers among different provinces in China, including International Peace Maternity and Child Health Hospital (Shanghai), Ningbo Women and Children’s Hospital (Zhejiang Province), Suzhou Municipal Hospital (Jiangsu Province). The study was approved by the research ethics board of each center and written informed consent forms (ICFs) were obtained from all the participants before inclusion.

Subjects were identified from the database in three centers from January 2014 to October 2019 using the following inclusion and exclusion criteria. The inclusion criteria were set as follows: 1) female participants aged between 20 and 45 years, 2) participants with serum AMH measurement within 12 months before undergoing IVF/ICSI cycles. The participants were excluded if they met the following criteria: 1) participants who underwent pre-implantation genetic testing (PGT), 2) participants using donor semen or donor oocyte, 3) mixed transfers with embryos retrieved from different oocyte retrieval cycles, 4) women with severe chronic diseases, 5) women for whom main data were missing or who were lost to follow-up. The participants were categorized into three groups according to the <25^th^(low), 25^th^ to 75^th^(average), and >75^th^(high) percentile of serum AMH concentration (0.01-1.76, 1.76-5.41, 5.41-25.00ng/ml). The subgroup analysis was conducted based on the number of live births.

### AMH measurement

Serum samples were collected from all participants and measured directly after arriving in the laboratory. In two of our study centers, the serum AMH was measured with chemiluminescent immunoassay (CLIA) by Kaeser 1000 chemiluminescence analyzer of Guangzhou Kangrun Biotechnology Co., Ltd. and its corresponding kit according to the manufacturer’s instructions. The intra-assay and inter-assay coefficient of the variation (CV%) was <8% and <15%. The limit of detection (LoD) was <0.06 ng/ml. And in the other study center, the electrochemiluminescence method with DXI800 chemiluminescence analyzer of Beckman Company and its corresponding kit was adopted for AMH measurement. The total CV% was <8% in the analytical measure range of 0.02 to 24 ng/ml, and the limit of detection was 0.02 ng/ml.

### IVF/ICSI procedures

The process of IVF or ICSI was conducted according to the standard protocols of our study centers. We performed different types of controlled ovarian hyperstimulation (COH) protocols (gonadotropin-releasing hormone (GnRH)-agonist protocol, GnRH-antagonist protocol, micro-flare protocol or others) according to the state of each patient (age, ovarian reserve and others). After COH, when the leading follicle reached 20mm in diameter or at least two follicles reached 18 mm, ovulation was induced by giving human chorionic gonadotropin (HCG) or gonadotropin-releasing hormone agonists (GnRH-a). Oocyte retrieval was performed 34-38 hours later and oocytes were fertilized by either conventional IVF or intracytoplasmic sperm injection after the assessment of semen quality. Subsequently, viable embryos were transferred in fresh embryo transfer cycles or frozen-thawed embryo transfer (FET) cycles after oocyte retrieval and routine corpus luteum support was performed after transplantation if conceived.

### Outcome measurements

Maternal baseline information was derived from the electronic database of the hospitals, including sociodemographic characteristics and reproductive history. We further abstracted the ART procedures and most of the perinatal outcomes from the database of the hospitals, while the neonatal morbidity and mortality were followed up and recorded by well-trained clinical personnel. The pregnancy outcomes assessed included hypertensive disorders in pregnancy (HDP), GDM, Intrahepatic cholestasis of pregnancy (ICP), placental abruption, placenta previa, oligohydramnios, premature rupture of membrane (PROM), postpartum hemorrhage (PPH) and mode of delivery. While neonatal outcomes were assessed including the gender of neonates, birth weight, preterm birth (PTB), weight for gestational age, neonatal infection, admission to the neonatal intensive care unit (NICU), neonatal asphyxia, neonatal jaundice, and congenital anomaly. Preterm birth was defined as delivery at less than 37 weeks, and very preterm was defined as delivery of baby between 28 and 32 gestational weeks of pregnancy. LGA or SGA was defined as a birth weight more than 90th centile or less than 10th centile of our population for a specific gestational age and sex, respectively (19, 20). Diagnoses were coded according to the International Classification of Diseases version 10(ICD-10).

### Statistical analysis

Continuous variables were presented as mean (standard deviation (SD)) or median (inter-quartile range) as appropriate. Comparisons of the continuous variables among three AMH groups were performed with the use of the Analysis of Variance (ANOVA) test or Kruskal-Wallis test. Categorical variables were represented as frequencies with proportions, while the Pearson Chi-square test or Fisher’s exact test was used to compare the distribution of demographics between categorical variables. Odds ratios (ORs) and 95% confidence intervals (CIs) were calculated using logistic regression to evaluate the association between serum AMH levels and each perinatal outcome following IVF/ICSI. To analyze the pregnancy and neonatal outcomes in singleton pregnancies, multinomial logistic regression was used to adjust ORs for potential confounding factors. While analyzing the neonatal outcomes of multiples, we performed multilevel logistic regression and adjusted for potential confounding factors (21). Those factors were selected according to baseline analysis and published literature.

The statistical analyses were performed using R software version 3.4.4 (R Foundation for Statistical Computing, Vienna, Austria). All of the statistical analyses were two-sided with a 5% level of significance.

## Results

**Figure 1:**
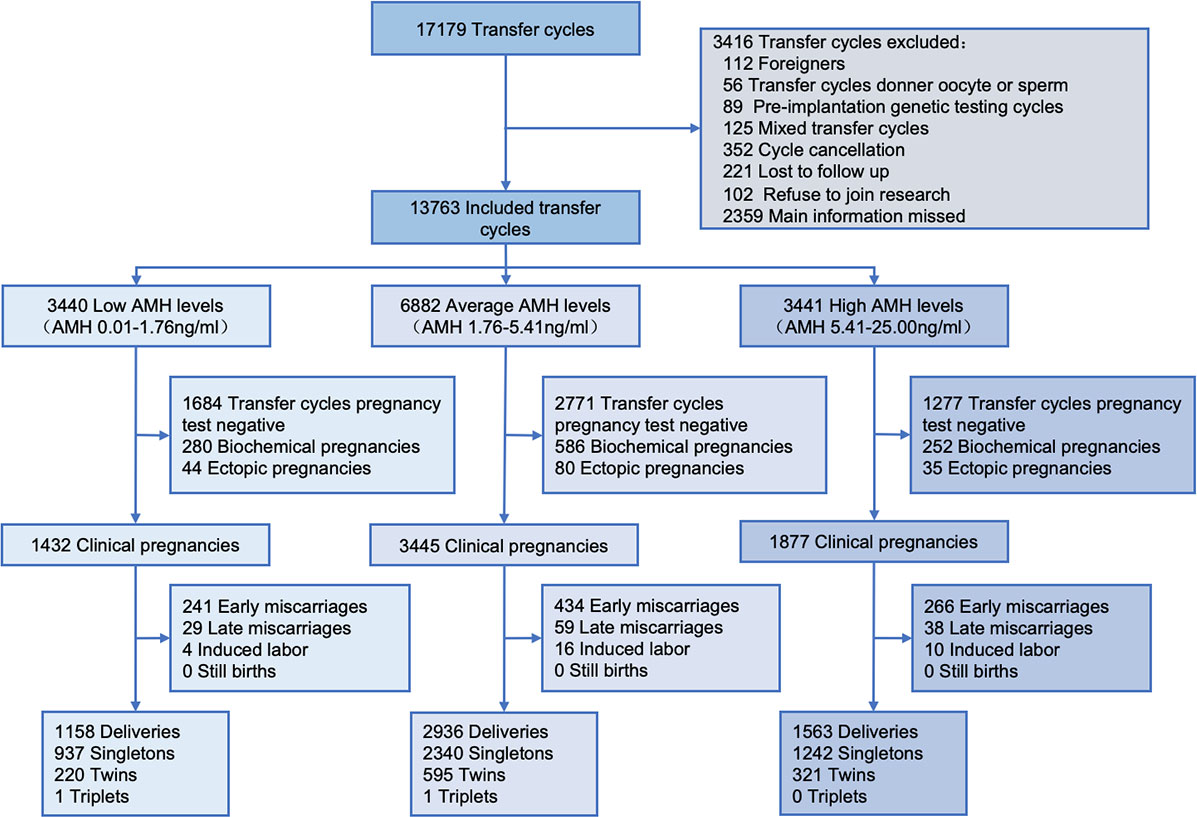
Flow chart of the study cohort.

The flowchart of the study cohort was shown in **Figure 1**. A total of 13,763 cycles met the eligibility criteria and were included in the cohort (3440 cycles in the low AMH group, 6882 cycles in the average AMH group, and 3441 cycles in the high AMH group). 5657 women with live-born babies (6797 live births with 4519 singletons and 1138 multiples) were further included in the analysis of perinatal outcomes.

The baseline characteristics of the participants with live birth deliveries stratified by AMH levels were presented in **Table 1**. Socio-demographic characteristics including pre-gestational BMI, education attainment, occupation and smoking status were similar among the three groups. However, the distribution of maternal age, paternal age and residence were different among groups (*p*<0.001). Significant differences were found in the race only in singleton delivery (*p*<0.001). Differences in the reproductive history of the participants were found in the parity, gravidity, duration of infertility, primary infertility, and causes of infertility (*p*<0.05), while no statistically significant differences were found between levels of AMH regarding times of abortion and history of ectopic pregnancy. In women with multiple deliveries, the history of ectopic pregnancy and causes of infertility were different among groups, the history of ectopic pregnancy is more frequent in women with low AMH levels. Additionally, gravidity, parity, times of abortion, and duration of infertility were comparable among the three groups. Characteristics of ART procedures (oocyte retrieval and embryo transfer cycles) according to AMH levels were presented in **Table S1**.

**Table 1:** Baseline characteristics of participants with live birth deliveries according to AMH levels.

	Singleton delivery				Multiple deliveries			
	Low AMH (N=937)	Average AMH (N=2340)	High AMH (N=1242)	*P* value	Low AMH (N=221)	Average AMH (N= 596)	High AMH (N=321)	*P* value
	n (%)	n (%)	n (%)		n (%)	n (%)	n (%)	
**Socio-demographic characteristics**								
**Maternal age (years)**	32.18±4.02	30.58±3.74	29.64±3.37	<0.001	31.45±3.53	29.68±3.36	29.89±3.40	<0.001
**Paternal age (years)**	33.78±5.29	32.25±4.78	31.24±4.38	<0.001	32.50±4.35	31.47±4.40	31.15±4.10	<0.001
**Pre-gestational BMI (kg/m2)**	22.02±2.95	21.98±3.00	22.04±3.06	0.897	22.29±3.27	22.06±2.84	22.05±2.99	0.814
**Race**								
Han	859 (98.5)	2153 (99.6)	1159 (99.7)	<0.001	206 (99.5)	562 (99.6)	304 (99.7)	0.956
Minority	13 (1.5)	9 (0.4)	3 (0.3)		1 (0.5)	2 (0.4)	1 (0.3)	
**Residence**								
Residents	830 (88.6)	2042 (87.3)	999 (80.4)	<0.001	194 (87.8)	523 (87.8)	249 (77.6)	<0.001
Immigrants/Nonresidents	107 (11.4)	298 (12.7)	243 (19.6)		27 (12.2)	73 (12.2)	72 (22.4)	
**Education attainment**								
Primary school or lower	21 (2.2)	37 (1.6)	15 (1.2)	0.415	4 (1.8)	9 (1.5)	5 (1.6)	0.98
Middle or high school	365 (39)	907 (38.8)	474 (38.3)		82 (37.4)	212 (35.8)	113 (35.2)	
Collage or above	550 (58.8)	1391 (59.6)	750 (60.5)		133 (60.7)	372 (62.7)	203 (63.2)	
**Occupation**								
Employed	629 (71.2)	1514 (70.8)	709 (69.1)	0.838	154 (72.3)	379 (69.5)	179 (70.8)	0.055
Self-employed	115 (13)	290 (13.6)	147 (14.3)		35 (16.4)	69 (12.7)	25 (9.9)	
Unemployed	139 (15.7)	333 (15.6)	170 (16.6)		24 (11.3)	97 (17.8)	49 (19.4)	
**Smoking status**								
No	75 (97.4)	240 (98.8)	251 (98.8)	0.625	12 (92.3)	59 (98.3)	80 (98.8)	0.288
Yes	2 (2.6)	3 (1.2)	3 (1.2)		1 (7.7)	1 (1.7)	1 (1.2)	
**History of reproduction**								
**Parity**								
0	473 (50.5)	1214 (51.9)	713 (57.4)	<0.001	128 (57.9)	383 (64.3)	211 (65.7)	0.236
1	223 (23.8)	609 (26.0)	302 (24.3)		57 (25.8)	120 (20.1)	70 (21.8)	
≥2	241 (25.7)	517 (22.1)	227 (18.3)		36 (16.3)	93 (15.6)	40 (12.5)	
**Gravidity**								
0	816 (87.1)	2106 (90)	1168 (94)	<0.001	207 (93.7)	569 (95.5)	305 (95.0)	0.673
1	114 (12.2)	218 (9.3)	69 (5.6)		14 (6.3)	25 (4.2)	15 (4.7)	
2	7 (0.7)	16 (0.7)	5 (0.4)		0 (0)	2 (0.3)	1 (0.3)	
**Number of previous abortions**								
0	606 (64.7)	1519 (64.9)	848 (68.3)	0.239	151 (68.3)	435 (73.0)	230 (71.7)	0.353
1-2	300 (32.0)	744 (31.8)	363 (29.2)		66 (29.9)	144 (24.2)	86 (26.8)	
≥3	31 (3.3)	77 (3.3)	31 (2.5)		4 (1.8)	17 (2.8)	5 (1.6)	
**Previous ectopic pregnancy**								
No	763 (81.4)	1917 (81.9)	1041 (83.8)	0.262	187 (84.6)	513 (86.1)	292 (91.0)	0.048
Yes	174 (18.6)	423 (18.1)	201 (16.2)		34 (15.4)	83 (13.9)	29 (9.0)	
**Duration of infertility (years)**								
1-2	265 (28.5)	642 (27.7)	349 (28.4)	0.015	56 (25.9)	175 (29.5)	79 (24.8)	0.588
3-4	287 (30.9)	851 (36.7)	447 (36.3)		83 (38.4)	223 (37.6)	125 (39.2)	
≥5	378 (40.6)	826 (35.6)	434 (35.3)		77 (35.6)	195 (32.9)	115 (36.1)	
**Primary infertility**								
No	464 (49.5)	1126 (48.1)	529 (42.6)	0.001	93 (42.1)	213 (35.7)	110 (34.3)	0.149
Yes	473 (50.5)	1214 (51.9)	713 (57.4)		128 (57.9)	383 (64.3)	211 (65.7)	
**Causes of infertility**								
Tubal infertility	262 (28.1)	563 (24.2)	289 (23.4)	<0.001	82 (37.3)	131 (22.2)	78 (24.4)	<0.001
PCOS	10 (1.1)	35 (1.5)	128 (10.4)		0 (0)	10 (1.7)	34 (10.6)	
Anovulation (not PCOS)	14 (1.5)	25 (1.1)	23 (1.9)		6 (2.7)	7 (1.2)	12 (3.8)	
Endometriosis	53 (5.7)	130 (5.6)	49 (4.0)		6 (2.7)	26 (4.4)	14 (4.4)	
Male-factor infertility	100 (10.7)	434 (18.7)	166 (13.4)		19 (8.6)	107 (18.1)	47 (14.7)	
Unexplained infertility	12 (1.3)	29 (1.2)	6 (0.5)		1 (0.5)	7 (1.2)	0 (0)	
Combined	483 (51.7)	1111 (47.7)	575 (46.5)		106 (48.2)	303 (51.3)	135 (42.2)	

AMH, anti-müllerian hormone; BMI, body mass index; PCOS, polycystic ovary syndrome. Variables containing missing data were retained in the analyses.

**Figure 2:**
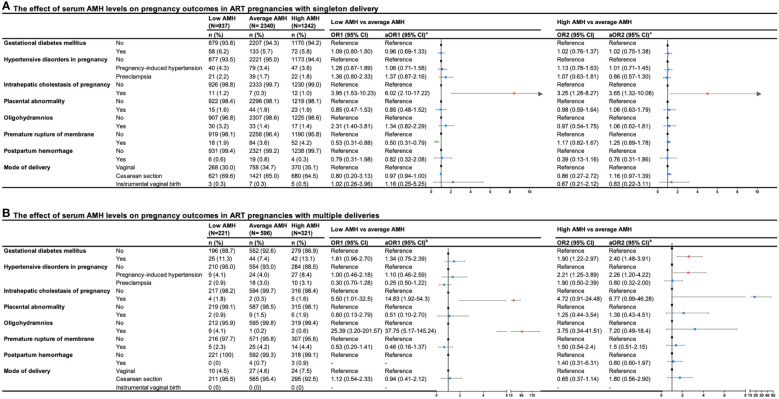
Forest plot summary of logistic regression analysis for risks of pregnancy outcomes in ART pregnancies with **(A)** singleton and **(B)** multiple deliveries. OR, odd ration; CI, confidence interval; aOR, adjusted odds ratio. aaOR was adjusted maternal age, paternal age, race, residence, gravidity, parity, duration of infertility, primary infertility, causes of infertility, study center, controlled ovarian stimulation protocol, type of insemination, transfer cycle types, embryo types, number of embryos transferred. baOR was adjusted maternal age, paternal age, residence, gravidity, parity, primary infertility, causes of infertility, study center, controlled ovarian stimulation protocol, type of insemination, transfer cycle types, embryo types.

The pregnancy outcomes of different groups stratified by serum AMH levels were shown in **Figure 2**, we presented the crude and adjusted odds ratios assessing the risks in the low AMH group and high AMH group compared with the average AMH group. After adjusting for confounding factors in logistic regression analyses, an increased risk of ICP was found to be associated with low and high levels of AMH in singleton delivery (aOR1 = 6.02, 95%CI: 2.10-17.22; aOR2 = 3.65, 95%CI: 1.32-10.08). In multiple deliveries, the low AMH group was also found to have an increased risk of ICP compared with the average AMH group (aOR1 = 14.83, 95%CI: 1.92-54.30). In addition, low levels of AMH compared to average levels of AMH were associated with a lower risk of PROM in women with singleton delivery (aOR1 = 0.50, 95%CI:0.31-0.79). Although not found in singleton delivery, high levels of AMH were associated with a higher risk of gestational diabetes mellitus and gestational hypertension in multiple deliveries (gestational diabetes mellitus: aOR2 = 2.40, 95%CI:1.48-3.91; gestational hypertension: aOR2 = 2.26, 95%CI: 1.20-4.22), while low levels of AMH were also associated with increased risk of oligohydramnios in women with multiple deliveries compared to average levels of AMH (aOR1 = 37.75, 95%CI: 5.17-145.24). There were no significant differences in risks regarding other pregnancy outcomes among the three groups.

**Figure 3:**
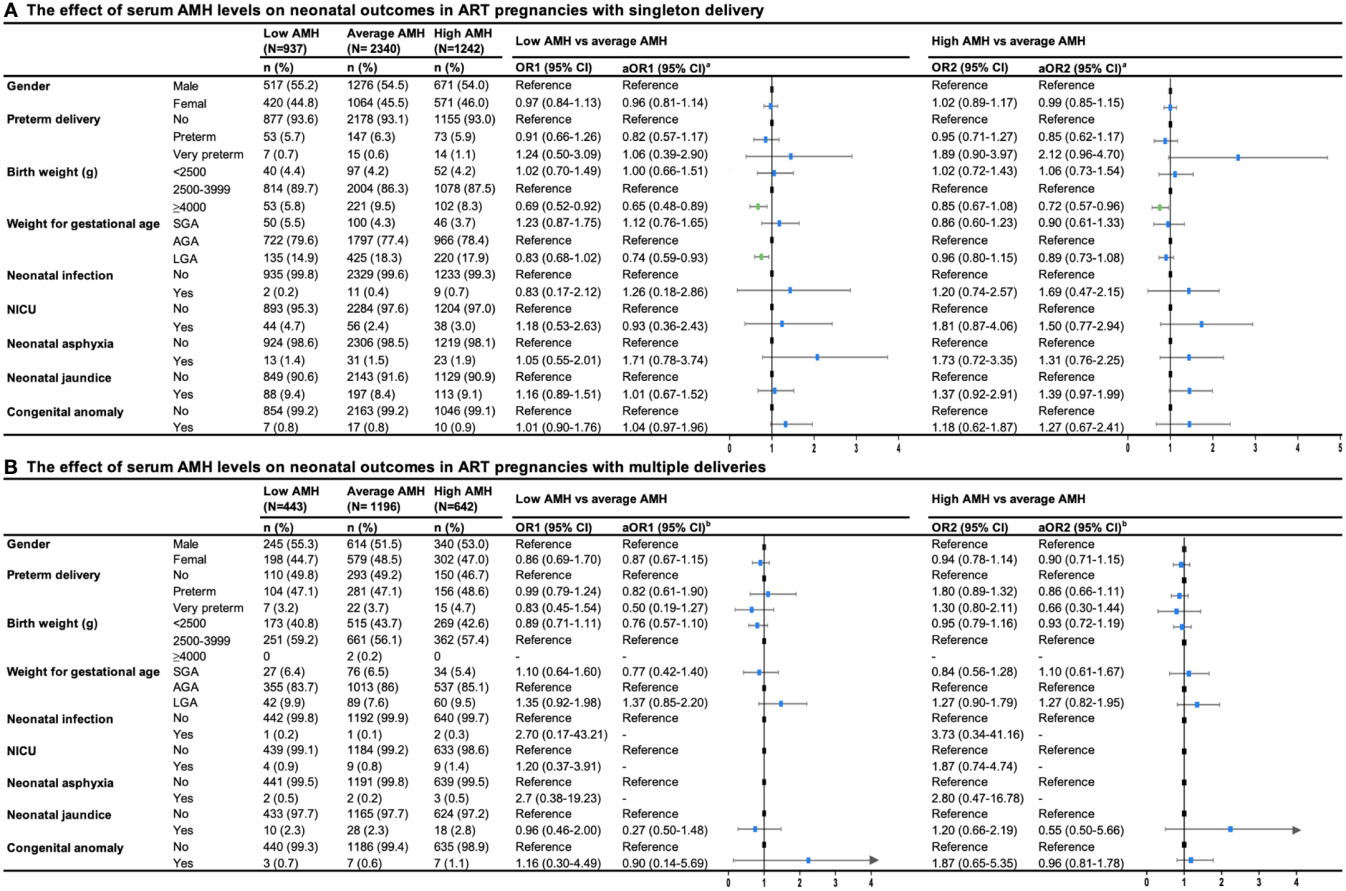
Forest plot summary of logistic regression analysis for risks of neonatal outcomes in ART pregnancies with **(A)** singleton and **(B)** multiple deliveries. OR, odds ration; CI, confidence interval; aOR, adjusted odds ration; SGA, small for gestational age; AGA, appropriate for gestational age; NICU, neonatal care unit. aaOR was adjusted for maternal age, paternal age, race, residence, gravidity, parity, duration of infertility, primary infertility, causes of infertility, study center, controlled ovarian stimulation protocol, type of insemination, transfer cycle types, embryo types, number of embryos transferred. baOR was adjusted maternal age, paternal age, residence, gravidity, parity, primary infertility, causes of infertility, study center, controlled ovarian stimulation protocol, type of insemination, transfer cycle types, embryo types.

**Figure 3** presented neonatal outcomes among three groups of serum AMH in women with singleton delivery and multiple deliveries. In singleton delivery, an decreased risk of macrosomia was found in the low AMH group compared with the average AMH group (aOR1 = 0.65, 95%CI: 0.48-0.89), while the high AMH group showed a similar effect (aOR2 = 0.72, 95%CI: 0.57-0.96). Additionally, there was an decreased risk of large for gestation age (LGA) in a group with lower levels of AMH compared with the average AMH group (aOR1 = 0.74, 95%CI: 0.59-0.93). There was no evidence of differences in preterm birth, congenital anomaly, and other neonatal complications among the three groups in both singleton delivery and multiple deliveries.

Further analyses were conducted in women with single embryo transfer and singleton live birth delivery. A total of 1985 cycles were included (341 fresh transfer cycles and 1644 frozen transfer cycles). The baseline and characteristics of ART procedures in fresh/frozen single embryo transfer cycles according to AMH levels were provided in **Tables S2** **,** **S3**. The effect of serum AMH levels on pregnancy and neonatal outcomes with fresh/frozen single embryo transfers were generally consistent with those of the primary analysis in singleton delivery, except that the risk of GDM increased in the low AMH group with fresh cycles, the risk of cesarean section decreased in the high AMH group with frozen cycles and the difference in the risk of ICP, macrosomia and LGA was no longer significant. Details are provided in **Tables S4** through **S7** in the **Supplementary Materials**.

## Discussion

In this multi-center retrospective cohort study of ART patients, we highlight in women with singleton delivery, low AMH levels increased the risk of ICP. There are also some protective factors, for instance, among women of singleton delivery, high AMH levels are associated with a lower risk of macrosomia as well as low levels of AMH are less likely to have PROM and LGA. Moreover, in women with multiple deliveries, we demonstrated that high levels of AMH increased the risk of ICP, GDM and PIH, while low AMH levels are associated with an increased risk of ICP and oligohydramnios. The findings of our study suggest an association between AMH and pregnancy outcomes among women undergoing IVF/ICSI.

The safety of ART procedures has long been a major concern among people who received the treatment, several meta-analyses of cohort studies have demonstrated adverse pregnancy outcomes among ART pregnancies, including GDM and PIH (16, 22). Although characteristics of infertility, advanced age and underlying polycystic ovary syndrome might result in confounders of the association, some prospective studies provide significant associations between ART and adverse pregnancy outcomes after adjusting for various confounders (23, 24). Thus, plasma markers as a screen for adverse outcomes are quite in need. Interestingly, AMH, a clinical marker of ovarian reserve, several studies have suggested its potential relation to specific pregnancy complications (such as preterm birth and PIH), while the relationship remains unclear concerning their limited sample size and conflicting results (10–12).

Transfer of multiple embryos in ART procedures used to bring a large number of multiple pregnancies and related risks in the last few years (25). Despite single-embryo transfer (SET) has been accepted as the best practice in clinical use, the ratio of twin delivery among total deliveries in ART was 27.9% in 2016(Chinese mainland) (13). Our study demonstrated that high AMH levels increased the risk of PIH and GDM in multiple deliveries after ART (Gestational hypertension: aOR2 = 2.26, 95% CI: 1.20-4.22; Gestational diabetes mellitus: aOR2 = 2.40, 95% CI: 1.48-3.91), low AMH levels increase the risk of oligohydramnios. Nonetheless, we failed to observe a similar association in singleton deliveries. Hypertensive disorder of pregnancy, which affect up to 10% of all pregnancies, is one of the leading causes of pregnancy-related deaths (26, 27). The relationship between AMH and HDP has been a controversial topic in recent studies. A case-control study conducted by Birdir et al. observed the median multiple of the expected median value of AMH was comparable between the PE (Preeclampsia) group and the controls (1.040, IQR 0.941–1.081 versus 0.995, IQR 0.939–1.065, p = 0.147), indicating AMH might not be a suitable marker for prediction of PE (28). However, several studies have observed that low levels of AMH are associated with a higher risk of HDP (10, 29). As for GDM, the association between AMH and GDM was not identified in previous studies (10). In the present study, we measure maternal AMH levels before pregnancy instead of measurement during pregnancy in other studies, which might result in the discrepancy. In addition, previous studies have not performed similar research in multiple deliveries. Mechanisms underlying the effects on pregnancy complications need more investigation. Detection of AMH receptors in cardiac tissue suggests the linkage of AMH with the circulatory system (30). Skałba et al. (31) documented that plasma AMH level is associated with insulin resistance (IR) both in PCOS (group) and control group, while Tokmak et al. (32) proved a similar correlation in non-obese adolescent females with PCOS. Considering IR is closely related to the development of GDM, AMH might play a role in the development of GDM. In summary, this study suggests that we should put more attention to abnormal AMH levels in women with multiple pregnancies. More specifically, abnormal AMH levels should be concerned when we determine the number of embryos transferred, single-embryo transfer is relatively more recommended.

Our study illustrates the association between abnormal AMH levels and ICP for the first time (low AMH levels are associated with increased risk of ICP in singleton and multiple deliveries). ICP is the most common hepatic disorder related to pregnancy, which usually develops within the third trimester of pregnancy and presents with pruritus as well as elevated levels of bile acid and/or alanine aminotransferase (33). Estrogen-bile acid axis was thought to play a dominant role in the pathogenesis of ICP (34), yet AMH was proved to decrease FSH-induced CYP19a1 expression, leading to reduced estradiol (E2) levels (1, 35), we could assume that the association between AMH and E2 might attribute to the effects of AMH on the risk of ICP. While the molecular mechanisms need more investigations.

Notably, through the analysis of neonatal outcomes, we also observed that circulating levels of AMH influence the risk of macrosomia and LGA in singleton deliveries, indicating some underlying nutritional and metabolic alterations in the offspring. An increasing number of studies have supported the theory of developmental origins of health and disease (DOHaD), which refers to the theory that predisposing factors to chronic diseases are established in early life, specifically by the intrauterine environment (36). Both human and animal studies have confirmed that the developing fetus is susceptible to *in-utero* exposures, including air pollution, high-fat diet and hyperglycemia (37, 38). Additionally, recent studies also demonstrated that high AMH levels *in utero* might induce metabolic and reproductive alterations in rodent animals, which suggested the potential effects of AMH on perinatal outcomes (39). Our results also demonstrated that AMH levels are not associated with the risk of preterm birth in women undergoing IVF/ICSI, which is consistent with a previous study that is also based on women undergoing IVF/ICSI cycles (40). However, recent studies suggested AMH level as a risk factor of preterm birth in PCOS patients (11, 12). The differences might attribute to the heterogeneity of the population thanks to the higher AMH level in PCOS patients compared with non-PCOS patients (41). Future studies including long-term follow-up studies are needed to illustrate the long-term effects and potential mechanisms.

The strengths of our study include the novelty as the first research to present the association between maternal AMH levels and pregnancy outcomes after ART, as well as the size of the cohort (largest to our knowledge). Additionally, maternal levels of AMH before pregnancy give us a more advanced vision to assess the risk of complications compared with measurement in the first or second trimester of pregnancy. Moreover, in this retrospective cohort study, the confounding factors were also adjusted for analysis, either previously reported to have effects on AMH levels or varied significantly among groups stratified by AMH. Despite the limited knowledge of the pathophysiology of AMH, we provide distinctive insights on its potential to be a marker of pregnancy outcomes. Similarly, we recognize that there are still limitations in our study. First, missing data regarding clinical and follow-up information was inevitable thanks to the retrospective cohort, which resulted in information bias. Second, the discrepancy of AMH measurement methods in different centers is also a source of bias, although study center was adjusted as a confounding factor in the logistic regression. Third, the relatively low morbidity restricts us to achieve a more accurate confidence interval, thus leading to limitations in our conclusions.

In conclusion, this is the first multi-center retrospective cohort study to indicate the association between maternal AMH levels and adverse perinatal outcomes in IVF/ICSI. Our results proved the potential role of AMH as a predictive marker for adverse pregnancy outcomes. Abnormal AMH levels increased the risk of ICP regardless of the number of live births, while high AMH levels are associated with risks of GDM and PIH only in women with multiple deliveries. In addition, AMH can also be used as a protective factor concerning PROM, macrosomia and LGA. Fortunately, serum AMH levels were not associated with adverse neonatal outcomes in IVF/ICSI. The findings of our study will extend the application of AMH during pregnancy and provide clinicians with some clues for practice. The association between high AMH levels and pregnancy complications among multiple pregnancies also supports the use of SET in these patients.

## Data availability statement

The raw data supporting the conclusions of this article will be made available by the authors, without undue reservation.

## Ethics statement

The studies involving human participants were reviewed and approved by The Institutional Review Board of the International Peace Maternal and Child Health Hospital. The patients/participants provided their written informed consent to participate in this study.

## Author contributions

H-FH, Y-TW and Y-CH designed the study concept. Y-CH and K-ZS conducted the statistical analysis and drafted the manuscript. Y-CH, Y-TW, JC and Q-XM were responsible for data collection and data curation. H-FH and Y-TW critically revised the manuscript. All authors contributed to the article and approved the submitted version.
