# Supplementary material for: Serum anti-Müllerian hormone levels are associated with perinatal outcomes in women undergoing IVF/ICSI: A multicenter retrospective cohort study
Source: Front Endocrinol (Lausanne). 2023 Feb 21;14:1081069. doi: 10.3389/fendo.2023.1081069 (PMC9990865; doi:10.3389/fendo.2023.1081069)
Supplement: Supplementary file 1 [file Table_1.docx]

**Supplementary material**

| **Table S1. Characteristics of ART procedures according to AMH levels.** | | | | | | | | | | | |
| --- | --- | --- | --- | --- | --- | --- | --- | --- | --- | --- | --- |
|  | **Singleton delivery** | | | |  | **Multiple deliveries** | | | | | |
|  | **Low AMH (N=937)** | **Average AMH**  **(N= 2340)** | **High AMH (N=1242)** | ***P* value** |  | **Low AMH (N=221)** | **Average AMH**  **(N= 596)** | | **High AMH (N=321)** | | ***P* value** |
|  | **n (%)** | **n (%)** | **n (%)** |  |  | **n (%)** | **n (%)** | | **n (%)** | |  |
| **Characteristics of oocytes retrieval cycle** |  |  |  |  |  |  |  | |  | |  |
| **COH protocol** |  |  |  |  |  |  |  | |  | |  |
| GnRH-agonist regimen | 477 (51.0) | 1706 (72.9) | 617 (49.7) | ＜0.001 |  | 132 (59.7) | 448 (75.3) | | 172 (53.6) | | ＜0.001 |
| GnRH-antagonist regimen | 240 (25.6) | 561 (24.0) | 605 (48.8) |  |  | 47 (21.3) | 133 (22.4) | | 142 (44.2) | |  |
| Microflare protocol | 116 (12.4) | 19 (0.8) | 5 (0.4) |  |  | 20 (9.0) | 4 (0.7) | | 5 (1.6) | |  |
| Others | 103 (11) | 54 (2.3) | 14 (1.1) |  |  | 22 (10.0) | 10 (1.7) | | 2 (0.6) | |  |
| **Duration of gonadotropin stimulation** | 9.23±2.84 | 10.07±2.14 | 10.05±2.38 | ＜0.001 |  | 9.08±2.62 | 10.24±2.20 | | 10.10±2.54 | | ＜0.001 |
| ≤10 days | 916 (98.5) | 2275 (97.8) | 1171 (95.8) | ＜0.001 |  | 218 (99.5) | 576 (97.8) | | 299 (95.2) | | 0.006 |
| ＞10 days | 14 (1.5) | 51 (2.2) | 51 (4.2) |  |  | 1 (0.5) | 13 (2.2) | | 15 (4.8) | |  |
| **Dose of gonadotropin (IU)** | 2217.9±1009.8 | 1846.4±790.7 | 1502.3±611.7 | ＜0.001 |  | 2069.4±824.5 | 1929.7±814.1 | | 1560.2±689.6 | | ＜0.001 |
| **Hormone level on the day of hCG trigger** |  |  |  |  |  |  |  | |  | |  |
| LH, Median (IQR) (IU/l) | 1.92 (1.35-2.78) | 1.95 (1.40-2.78) | 1.62 (1.16-2.70) | 0.308 |  | 2 (1.50-2.74) | 1.82 (1.42-2.22) | | 1.82 (1.02-2.43) | | 0.218 |
| E2, Median (IQR) (×103pmol/L) | 3.91 (2.50-5.83) | 7.16 (4.55-12.47) | 15.67 (8.21-18.12) | ＜0.001 |  | 4.19 (2.003-5.69) | 6.2 (4.46-10.95) | | 13.3 (7.02-18.12) | | ＜0.001 |
| P, Median (IQR) (nmol/l) | 0.94 (0.71-1.29) | 0.91 (0.69-1.26) | 1.09 (0.85-1.51) | ＜0.001 |  | 0.95 (0.66-1.15) | 1.13 (0.78-1.38) | | 1.09 (0.80-1.25) | | 0.405 |
| **Number of previous ART procedures** |  |  |  |  |  |  |  | |  | |  |
| 0 | 768 (85) | 2104 (91.4) | 1176 (95.8) | ＜0.001 |  | 175 (85.8) | 530 (91.7) | | 304 (96.5) | | ＜0.001 |
| 1 | 120 (13.3) | 173 (7.5) | 45 (3.7) |  |  | 24 (11.8) | 42 (7.3) | | 10 (3.2) | |  |
| ≥2 | 15 (1.7) | 25 (1.1) | 7 (0.6) |  |  | 5 (2.5) | 6 (1.0) | | 1 (0.3) | |  |
| **Type of insemination** |  |  |  |  |  |  |  | |  | |  |
| IVF | 665 (73.0) | 1585 (68.0) | 867 (70.1) | 0.002 |  | 174 (79.8) | 397 (66.8) | | 219 (68.9) | | ＜0.001 |
| ICSI | 246 (27.0) | 746 (32.0) | 370 (29.9) |  |  | 44 (20.2) | 197 (33.2) | | 99 (31.1) | |  |
| **Number of oocytes retrieved** | 7.25±4.59 | 11.48±5.19 | 15.36±6.83 | ＜0.001 |  | 7.52±4.29 | 10.97±4.70 | | 15.55±7.17 | | ＜0.001 |
| ≤10 | 695 (81.9) | 1085 (47.9) | 328 (27) | ＜0.001 |  | 158 (82.7) | 300 (53.1) | | 89 (28.8) | | ＜0.001 |
| 11-20 | 140 (16.5) | 1056 (46.6) | 665 (54.6) |  |  | 30 (15.7) | 242 (42.8) | | 158 (51.1) | |  |
| ＞20 | 14 (1.6) | 126 (5.6) | 224 (18.4) |  |  | 3 (1.6) | 23 (4.1) | | 62 (20.1) | |  |
| **Number of fertilized embryos** | 5.93±3.94 | 9.10±4.47 | 11.91±5.74 | ＜0.001 |  | 6.29±3.78 | 8.64±4.00 | | 12.04±6.02 | | ＜0.001 |
| **Characteristics of embryo transfer cycle** |  |  |  |  |  |  |  | |  | |  |
| **Transfer cycle types** |  |  |  |  |  |  |  | |  | |  |
| Fresh | 371 (39.6) | 725 (31.0) | 177 (14.3) | ＜0.001 |  | 114 (51.6) | 262 (44) | | 64 (19.9) | | ＜0.001 |
| Frozen | 566 (60.4) | 1615 (69.0) | 1065 (85.7) |  |  | 107 (48.4) | 334 (56) | | 257 (80.1) | |  |
| **Type of endometrium preparation (in FET cycle)** |  |  |  |  |  |  |  | |  | |  |
| Natural cycle | 249 (44.1) | 703 (43.5) | 275 (25.8) | ＜0.001 |  | 48 (44.9) | 143 (42.8) | | 77 (30.0) | | 0.002 |
| OS cycle | 4 (0.7) | 42 (2.6) | 51 (4.8) |  |  | 0 | 15 (4.5) | | 15 (5.8) | |  |
| HRT cycle | 312 (55.2) | 870 (53.9) | 739 (69.4) |  |  | 59 (55.1) | 176 (52.7) | | 165 (64.2) | |  |
| **Embryo types** |  |  |  |  |  |  |  | |  | |  |
| blastomere | 668 (71.3) | 1337 (57.1) | 693 (55.8) | ＜0.001 |  | 204 (92.3) | 552 (92.6) | | 287 (89.4) | | 0.227 |
| blastocyst | 269 (28.7) | 1003 (42.9) | 549 (44.2) |  |  | 17 (7.7) | 44 (7.4) | | 34 (10.6) | |  |
| **Number of embryos transferred** |  |  |  |  |  |  |  | |  | |  |
| 1 | 329 (35.1) | 1084 (46.3) | 572 (46.1) | ＜0.001 |  | 3 (1.4) | 19 (3.2) | | 12 (3.7) | | 0.332 |
| 2 | 602 (64.2) | 1248 (53.3) | 667 (53.7) |  |  | 218 (98.6) | 573 (96.1) | | 308 (96) | |  |
| 3 | 6 (0.6) | 8 (0.3) | 3 (0.2) |  |  | 0 | 4 (0.7) | | 1 (0.3) | |  |
| **Endometrial thickness on the day of embryo transfer (mm)** | 9.61±2.07 | 9.43±1.84 | 9.19±1.68 | ＜0.001 |  | 9.76±2.08 | 11.39±4.20 | | 9.41±1.71 | | 0.164 |
| *Note:* AMH, anti-müllerian hormone; COH, controlled ovarian stimulation; GnRH, gonadotropin-releasing hormone; hCG, human chorionic gonadotropin; IQR, interquartile range; LH, luteinizing hormone; E2, estradiol; P, progesterone; ART, assisted reproductive technology; IVF, in vitro fertilization; ICSI, intracytoplasmic sperm injection; FET, frozen embryo transfer; OS, ovarian stimulation; HRT, hormonal replacement therapy; Variables containing missing data were retained in the analyses. | | | | | | | |  | |  | |

| **TableS2. Baseline characteristics of participants with** **single embryo transfer in fresh/frozen cycles according to AMH levels.** | | | | | | | | | |
| --- | --- | --- | --- | --- | --- | --- | --- | --- | --- |
|  | **Fresh cycles** | | | |  | **Frozen cycles** | | | |
|  | **Low AMH (N=74)** | **Average AMH**  **(N= 213)** | **High AMH (N=54)** | **P value** |  | **Low AMH (N=255)** | **Average AMH**  **(N= 871)** | **High AMH (N=518)** | ***P* value** |
|  | **n (%)** | **n (%)** | **n (%)** |  |  | **n (%)** | **n (%)** | **n (%)** |  |
| **Socio-demographic characteristics** |  |  |  |  |  |  |  |  |  |
| **Maternal age (****years)** | 32 ± 4 | 30 ± 4 | 29 ± 2 | <0.001 |  | 32 ± 4 | 31 ± 4 | 30 ± 3 | <0.001 |
| **Paternal age (years)** | 34 ± 5 | 32 ± 5 | 30 ± 4 | <0.001 |  | 34 ± 6 | 32 ± 5 | 31 ± 4 | <0.001 |
| **Pre-gestational BMI (kg/m2)** | 21 (19, 23) | 21 (20, 23) | 21 (20, 24) | 0.465 |  | 22 (20, 24) | 21 (20, 23) | 22 (20, 24) | 0.090 |
| **Race** |  |  |  |  |  |  |  |  |  |
| Han | 68 (98.6) | 195 (100.0) | 48 (100.0) | 0.220 |  | 218 (98.6) | 792 (99.9) | 476 (100.0) | 0.016 |
| Minority | 1 (1.4) | 0 (0.0) | 0 (0.0) |  |  | 3 (1.4) | 1 (0.1) | 0 (0.0) |  |
| **Residence** |  |  |  |  |  |  |  |  |  |
| Residents | 66 (89.2) | 195 (91.5) | 49 (90.7) | 0.830 |  | 228 (89.4) | 785 (90.1) | 451 (87.1) | 0.206 |
| Immigrants/Nonresidents | 8 (10.8) | 18 (8.5) | 5 (9.3) |  |  | 27 (10.6) | 86 (9.9) | 67 (12.9) |  |
| **Education attainment** |  |  |  |  |  |  |  |  |  |
| Primary school or lower | 2 (2.7) | 8 (3.8) | 1 (1.9) | 0.583 |  | 5 (2.0) | 11 (1.3) | 3 (0.6) | 0.266 |
| Middle or high school | 22 (29.7) | 76 (35.7) | 23 (42.6) |  |  | 111 (43.5) | 365 (42.0) | 202 (39.1) |  |
| Collage or above | 50 (67.6) | 129 (60.6) | 30 (55.6) |  |  | 139 (54.5) | 494 (56.8) | 312 (60.3) |  |
| **Occupation** |  |  |  |  |  |  |  |  |  |
| Employed | 54 (73.0) | 146 (69.5) | 39 (75.0) | 0.828 |  | 180 (73.2) | 598 (71.4) | 326 (67.8) | 0.169 |
| Self-employed | 7 (9.5) | 29 (13.8) | 5 (9.6) |  |  | 23 (9.3) | 115 (13.7) | 73 (15.2) |  |
| Unemployed | 13 (17.6) | 35 (16.7) | 8 (15.4) |  |  | 43 (17.5) | 124 (14.8) | 82 (17.0) |  |
| **Smoking status** |  |  |  |  |  |  |  |  |  |
| No | 1 (100.0) | 4 (100.0) | 2 (100.0) | / |  | 13 (100.0) | 39 (100.0) | 46 (97.9) | 0.472 |
| Yes | 0 (0.0) | 0 (0.0) | 0 (0.0) |  |  | 0 (0.0) | 0 (0.0) | 1 (2.1) |  |
| **History of reproduction** |  |  |  |  |  |  |  |  |  |
| **Parity** |  |  |  |  |  |  |  |  |  |
| 0 | 35 (47.3) | 101 (47.4) | 33 (61.1) | 0.473 |  | 107 (42.0) | 431 (49.5) | 284 (54.8) | <0.001 |
| 1 | 21 (28.4) | 57 (26.8) | 11 (20.4) |  |  | 58 (22.7) | 228 (26.2) | 133 (25.7) |  |
| ≥2 | 18 (24.3) | 55 (25.8) | 10 (18.5) |  |  | 90 (35.3) | 212 (24.3) | 101 (19.5) |  |
| **Gravidity** |  |  |  |  |  |  |  |  |  |
| 0 | 65 (87.8) | 177 (83.1) | 49 (90.7) | 0.571 |  | 202 (79.2) | 763 (87.6) | 474 (91.5) | <0.001 |
| 1 | 8 (10.8) | 32 (15.0) | 4 (7.4) |  |  | 50 (19.6) | 102 (11.7) | 41 (7.9) |  |
| 2 | 1 (1.4) | 4 (1.9) | 1 (1.9) |  |  | 3 (1.2) | 6 (0.7) | 3 (0.6) |  |
| **Number of previous abortions** |  |  |  |  |  |  |  |  |  |
| 0 | 48 (64.9) | 151 (70.9) | 38 (70.4) | 0.695 |  | 151 (59.2) | 570 (65.4) | 361 (69.7) | 0.072 |
| 1-2 | 22 (29.7) | 57 (26.8) | 15 (27.8) |  |  | 92 (36.1) | 269 (30.9) | 141 (27.2) |  |
| ≥3 | 4 (5.4) | 5 (2.3) | 1 (1.9) |  |  | 12 (4.7) | 32 (3.7) | 16 (3.1) |  |
| **Previous ectopic pregnancy** |  |  |  |  |  |  |  |  |  |
| No | 61 (82.4) | 160 (75.1) | 47 (87.0) | 0.107 |  | 201 (78.8) | 690 (79.2) | 426 (82.2) | 0.337 |
| Yes | 13 (17.6) | 53 (24.9) | 7 (13.0) |  |  | 54 (21.2) | 181 (20.8) | 92 (17.8) |  |
| **Duration of infertility (years)** |  |  |  |  |  |  |  |  |  |
| 1-2 | 21 (28.8) | 71 (34.1) | 14 (26.4) | 0.771 |  | 70 (27.7) | 223 (25.9) | 139 (27.0) | 0.473 |
| 3-4 | 27 (37.0) | 73 (35.1) | 19 (35.8) |  |  | 82 (32.4) | 297 (34.5) | 193 (37.5) |  |
| ≥5 | 25 (34.2) | 64 (30.8) | 20 (37.7) |  |  | 101 (39.9) | 342 (39.7) | 182 (35.4) |  |
| **Primary infertility** |  |  |  |  |  |  |  |  |  |
| No | 39 (52.7) | 112 (52.6) | 21 (38.9) | 0.180 |  | 148 (58.0) | 440 (50.5) | 234 (45.2) | 0.003 |
| Yes | 35 (47.3) | 101 (47.4) | 33 (61.1) |  |  | 107 (42.0) | 431 (49.5) | 284 (54.8) |  |
| **Causes of infertility** |  |  |  |  |  |  |  |  |  |
| Tubal infertility | 15 (20.3) | 41 (19.3) | 10 (18.5) | 0.116 |  | 63 (24.8) | 185 (21.3) | 93 (18.1) | <0.001 |
| PCOS | 0 (0.0) | 0 (0.0) | 3 (5.6) |  |  | 3 (1.2) | 13 (1.5) | 47 (9.1) |  |
| Anovulation (not PCOS) | 2 (2.7) | 3 (1.4) | 0 (0.0) |  |  | 1 (0.4) | 11 (1.3) | 7 (1.4) |  |
| Endometriosis | 3 (4.1) | 9 (4.2) | 3 (5.6) |  |  | 10 (3.9) | 51 (5.9) | 18 (3.5) |  |
| Male-factor infertility | 7 (9.5) | 35 (16.5) | 5 (9.3) |  |  | 25 (9.8) | 159 (18.3) | 67 (13.0) |  |
| Unexplained infertility | 1 (1.4) | 1 (0.5) | 0 (0.0) |  |  | 4 (1.6) | 12 (1.4) | 3 (0.6) |  |
| Combined | 46 (62.2) | 123 (58.0) | 33 (61.1) |  |  | 148 (58.3) | 436 (50.3) | 279 (54.3) |  |
| *Note:* AMH, anti-müllerian hormone; BMI, body mass index; PCOS, polycystic ovary syndrome. Variables containing missing data were retained in the analyses. | | | | | | | | | |

| **Table S3. Characteristics of ART procedures in fresh/frozen single embryo transfer cycles according to AMH levels.** | | | | | | | | | |
| --- | --- | --- | --- | --- | --- | --- | --- | --- | --- |
|  | **Fresh cycles** | | | |  | **Frozen cycles** | | | |
|  | **Low AMH (N=74)** | **Average AMH**  **(N= 213)** | **High AMH (N=54)** | ***P* value** |  | **Low AMH (N=255)** | **Average AMH**  **(N= 871)** | **High AMH (N=518)** | ***P* value** |
|  | **n (%)** | **n (%)** | **n (%)** |  |  | **n (%)** | **n (%)** | **n (%)** |  |
| **COH protocol** |  |  |  |  |  |  |  |  |  |
| GnRH-agonist regimen | 45 (60.8) | 171 (80.3) | 28 (51.9) | <0.001 |  | 104 (40.8) | 644 (73.9) | 268 (51.8) | <0.001 |
| GnRH-antagonist regimen | 23 (31.1) | 41 (19.2) | 25 (46.3) |  |  | 63 (24.7) | 200 (23.0) | 240 (46.4) |  |
| Microflare protocol | 3 (4.1) | 0 (0.0) | 0 (0.0) |  |  | 36 (14.1) | 5 (0.6) | 2 (0.4) |  |
| Others | 3 (4.1) | 1 (0.5) | 1 (1.9) |  |  | 52 (20.4) | 22 (2.5) | 7 (1.4) |  |
| **Number of previous ART procedures** |  |  |  |  |  |  |  |  |  |
| 0 | 49 (71.0) | 173 (82.8) | 49 (71.0) | 0.109 |  | 221 (86.7) | 825 (94.7) | 507 (97.9) | <0.001 |
| 1 | 19 (27.5) | 30 (14.4) | 19 (27.5) |  |  | 31 (12.2) | 40 (4.6) | 11 (2.1) |  |
| ≥2 | 1 (1.4) | 6 (2.9) | 1 (1.4) |  |  | 3 (1.2) | 6 (0.7) | 0 (0.0) |  |
| **Type of insemination** |  |  |  |  |  |  |  |  |  |
| IVF | 59 (79.7) | 152 (71.4) | 35 (64.8) | 0.163 |  | 172 (68.8) | 565 (65.2) | 335 (64.9) | 0.519 |
| ICSI | 15 (20.3) | 61 (28.6) | 19 (35.2) |  |  | 78 (31.2) | 302 (34.8) | 181 (35.1) |  |
| **Number of oocytes retrieved** | 6 ± 4 | 10 ± 4 | 11 ± 4 | <0.001 |  | 8 ± 5 | 12 ± 5 | 15 ± 6 | <0.001 |
| ≤10 | 58 (87.9) | 119 (57.2) | 20 (37.7) | <0.001 |  | 197 (79.4) | 355 (40.9) | 118 (22.9) | <0.001 |
| 11-20 | 7 (10.6) | 87 (41.8) | 33 (62.3) |  |  | 48 (19.4) | 448 (51.7) | 319 (61.8) |  |
| ＞20 | 1 (1.5) | 2 (1.0) | 0 (0.0) |  |  | 3 (1.2) | 64 (7.4) | 79 (15.3) |  |
| **Number of fertilized embryos** | 4 ± 3 | 8 ± 4 | 8 ± 4 | <0.001 |  | 6 ± 4 | 10 ± 4 | 12 ± 5 | <0.001 |
| **Type of endometrium preparation (in FET cycle)** |  |  |  |  |  |  |  |  |  |
| Natural cycle | / | / | / | / |  | 124 (48.6) | 386 (44.3) | 141 (27.2) | <0.001 |
| OS cycle | / | / | / |  |  | 0 (0.0) | 24 (2.8) | 21 (4.1) |  |
| HRT cycle | / | / | / |  |  | 131 (51.4) | 461 (52.9) | 356 (68.7) |  |
| **Embryo types** |  |  |  |  |  |  |  |  |  |
| blastomere | 48 (64.9) | 80 (37.6) | 24 (44.4) | <0.001 |  | 43 (16.9) | 62 (7.1) | 39 (7.5) | <0.001 |
| blastocyst | 26 (35.1) | 133 (62.4) | 30 (55.6) |  |  | 212 (83.1) | 809 (92.9) | 479 (92.5) |  |
| **Endometrial thickness on the day of embryo transfer (mm)** | 9 (8, 10) | 10 (8, 11) | 9 (8, 11) | 0.140 |  | 9 (8, 10) | 9 (8, 10) | 9 (8, 10) | 0.679 |
| *Note:* AMH, anti-müllerian hormone; COH, controlled ovarian stimulation; GnRH, gonadotropin-releasing hormone; hCG, human chorionic gonadotropin; ART, assisted reproductive technology; IVF, in vitro fertilization; ICSI, intracytoplasmic sperm injection; FET, frozen embryo transfer; OS, ovarian stimulation; HRT, hormonal replacement therapy; Variables containing missing data were retained in the analyses. | | | | | | | | | |

|  |  |  |  |  | |  |  |  |  |  |  |
| --- | --- | --- | --- | --- | --- | --- | --- | --- | --- | --- | --- |
|  |  |  |  |  | |  |  |  |  |  |  |
| **T****able S4. The effect of serum AMH levels on pregnancy outcomes in ART pregnancies** **with** **fresh single embryo transfers.** | | | | | | | | | | |  |
|  | **Low AMH (N=74)** | **Average AMH (N= 213)** | **High AMH (N=54)** |  | **Low AMH vs average AMH** | | |  | **High AMH vs average AMH** | |  |
|  | **n (%)** | **n (%)** | **n (%)** |  | **OR1 (95% CI)** | | **aOR1 (95% CI)^a^** |  | **OR2 (95% CI)** | **aOR2 (95% CI) ^a^** | |
| **Gestational diabetes mellitus** |  |  |  |  |  | |  |  |  |  |  |
| No | 68 (91.9) | 204 (95.8) | 52 (96.3) |  | Reference | | Reference |  | Reference | Reference |  |
| Yes | 6 (8.1) | 9 (4.2) | 2 (3.7) |  | 2.00 (0.69-5.82) | | 4.14 (1.24-13.80) |  | 0.87 (0.18-4.16) | 0.76 (0.14-4.04) |  |
| **Hypertensive disorders in pregnancy** |  |  |  |  |  | |  |  |  |  |  |
| No | 70 (94.6) | 202 (94.8) | 54 (100.0) |  | Reference | | Reference |  | Reference | Reference |  |
| Pregnancy-induced hypertension | 4 (5.4) | 7 (3.3) | 0 (0.0) |  | 1.65 (0.47-5.80) | | 0.96 (0.21-4.39) |  | - | - |  |
| Preeclampsia | 0 (0.0) | 4 (1.9) | 0 (0.0) |  | - | | - |  | - | - |  |
| **Intrahepatic cholestasis of pregnancy** |  |  |  |  |  | |  |  |  |  |  |
| No | 73 (98.6) | 212 (99.5) | 54 (100.0) |  | Reference | | Reference |  | Reference | Reference |  |
| Yes | 1 (1.4) | 1 (0.5) | 0 (0.0) |  | 2.90 (0.18-47.02) | | 1.66 (0.08-32.75) |  | - | - |  |
| **Placental abnormality** |  |  |  |  |  | |  |  |  |  |  |
| No | 73 (98.6) | 208 (97.7) | 52 (96.3) |  | Reference | | Reference |  | Reference | Reference |  |
| Yes | 1 (1.4) | 5 (2.3) | 2 (3.7) |  | 0.57 (0.07-4.96) | | 0.88 (0.09-8.38) |  | 1.60 (0.30-8.48) | 1.22 (0.20-7.32) |  |
| **Oligohydramnios** |  |  |  |  |  | |  |  |  |  |  |
| No | 73 (98.6) | 211 (99.1) | 53 (98.1) |  | Reference | | Reference |  | Reference | Reference |  |
| Yes | 1 (1.4) | 2 (0.9) | 1 (1.9) |  | 1.45 (0.13-16.17) | | 1.98 (0.16-24.94) |  | 1.99 (0.18-22.37) | 1.85 (0.15-22.91) |  |
| **Premature rupture of membrane** |  |  |  |  |  | |  |  |  |  |  |
| No | 74 (100.0) | 205 (96.2) | 50 (92.6) |  | Reference | | Reference |  | Reference | Reference |  |
| Yes | 0 (0.0) | 8 (3.8) | 4 (7.4) |  | - | | - |  | 2.05 (0.59-7.08) | 2.21 (0.58-8.34) |  |
| **Mode of delivery** |  |  |  |  |  | |  |  |  |  |  |
| Vaginal | 28 (37.8) | 97 (46.0) | 20 (37.0) |  | Reference | | Reference |  | Reference | Reference |  |
| Cesarean section | 45 (60.8) | 112 (53.1) | 34 (63.0) |  | 1.39 (0.81-2.40) | | 1.36 (0.74-2.51) |  | 1.47 (0.80-2.72) | 1.75 (0.91-3.39) |  |
| Instrumental vaginal birth | 1 (1.4) | 2 (0.9) | 0 (0.0) |  | 1.73 (0.15-19.75) | | 1.42 (0.11-18.09) |  | - | - |  |
|  |  |  |  |  |  | |  |  |  |  |  |
| Note: OR, odds ratio; CI, confidence interval; aOR, adjusted odds ratio; | | | | | | | | | | |  |
| **^a^** aOR was adjusted for maternal age, paternal age, study center, controlled ovarian stimulation protocols, embryo types. | | | | | | | | | | |  |
|  |  |  |  |  | |  |  |  |  |  |  |
| **Table S5. The effect of serum AMH levels on neonatal outcomes in ART pregnancies with fresh single embryo transfers.** | | | | | | | | | | |  |
|  | **Low AMH (N=74)** | **Average AMH (N= 213)** | **High AMH (N=54)** |  | | **Low AMH vs average AMH** | |  | **High AMH vs average AMH** | |  |
|  | **n (%)** | **n (%)** | **n (%)** |  | | **OR1 (95% CI)** | **aOR1 (95% CI) ^a^** |  | **OR2 (95% CI)** | **aOR2 (95% CI) ^a^** |  |
| **Gender** |  |  |  |  | |  |  |  |  |  |  |
| Male | 44 (59.5) | 126 (59.2) | 37 (68.5) |  | | Reference | Reference |  | Reference | Reference |  |
| Female | 30 (40.5) | 87 (40.8) | 17 (31.5) |  | | 0.99 (0.58-1.69) | 0.97 (0.54-1.75) |  | 0.67 (0.35-1.26) | 0.59 (0.30-1.16) |  |
| **Preterm delivery** |  |  |  |  | |  |  |  |  |  |  |
| No | 72 (97.3) | 199 (93.4) | 50 (92.6) |  | | Reference | Reference |  | Reference | Reference |  |
| Preterm | 2 (2.7) | 12 (5.6) | 3 (5.6) |  | | 0.39 (0.09-1.78) | 0.66 (0.14-3.16) |  | 1.14 (0.36-3.60) | 0.87 (0.25-3.03) |  |
| Very preterm | 0 (0.0) | 2 (0.9) | 1 (1.9) |  | | - | - |  | - | - |  |
| **Birth weight (g)** | 3400 (3100, 3600) | 3300 (3050, 3600) | 3200 (3000, 3500) | |  | |  |  |  |  |  |
| <2500 | 3 (4.2) | 8 (3.8) | 3 (5.6) |  | | 1.07 (0.28-4.15) | 1.42 (0.33-6.11) |  | 1.44 (0.37-5.63) | 0.91 (0.21-3.98) |  |
| 2500-3999 | 66 (91.7) | 188 (88.3) | 49 (90.7) |  | | Reference | Reference |  | Reference | Reference |  |
| ≥4000g | 3 (4.2) | 17 (8.0) | 2 (3.7) |  | | 0.50 (0.14-1.77) | 0.38 (0.10-1.52) |  | 0.45 (0.10-2.02) | 0.37 (0.08-1.74) |  |
| **Weight for gestational age** |  |  |  |  | |  |  |  |  |  |  |
| SGA | 4 (5.6) | 7 (3.3) | 4 (7.4) |  | | 1.70 (0.48-6.03) | 1.74 (0.44-6.81) |  | 2.35 (0.66-8.41) | 2.32 (0.59-9.10) |  |
| AGA | 58 (80.6) | 173 (81.2) | 42 (77.8) |  | | Reference | Reference |  | Reference | Reference |  |
| LGA | 10 (13.9) | 33 (15.5) | 8 (14.8) |  | | 0.90 (0.42-1.95) | 0.87 (0.38-1.97) |  | 1.00 (0.43-2.32) | 0.96 (0.40-2.32) |  |
| **Neonatal infection** |  |  |  |  | |  |  |  |  |  |  |
| No | 5 (83.3) | 9 (100.0) | 3 (100.0) |  | | Reference | Reference |  | Reference | Reference |  |
| Yes | 1 (16.7) | 0 (0.0) | 0 (0.0) |  | | - | - |  | - | - |  |
| **NICU** |  |  |  |  | |  |  |  |  |  |  |
| No | 5 (83.3) | 9 (100.0) | 3 (100.0) |  | | Reference | Reference |  | Reference | Reference |  |
| Yes | 1 (16.7) | 0 (0.0) | 0 (0.0) |  | | - | - |  | - | - |  |
| **Neonatal asphyxia** |  |  |  |  | |  |  |  |  |  |  |
| No | 6 (75.0) | 9 (90.0) | 3 (75.0) |  | | Reference | Reference |  | Reference | Reference |  |
| Yes | 2 (25.0) | 1 (10.0) | 1 (25.0) |  | | 3.00 (0.22-40.93) | - |  | 3.00 (0.14-64.25) | - |  |
| **Neonatal jaundice** |  |  |  |  | |  |  |  |  |  |  |
| No | 3 (50.0) | 8 (88.9) | 2 (66.7) |  | | Reference | Reference |  | Reference | Reference |  |
| Yes | 3 (50.0) | 1 (11.1) | 1 (33.3) |  | | 8.00 (0.58-110.26) | - |  | 3.00 (0.14-64.25) | - |  |
| **Congenital anomaly** |  |  |  |  | |  |  |  |  |  |  |
| No | 71 (98.6) | 211 (99.5) | 54 (100.0) |  | | Reference | Reference |  | Reference | Reference |  |
| Yes | 1 (1.4) | 1 (0.5) | 0 (0.0) |  | | 2.97 (0.18-48.13) | 3.23 (0.16-64.27) |  | - | - |  |
|  |  |  |  |  | |  |  |  |  |  |  |
| *Note:* OR, odds ratio; CI, confidence interval; aOR, adjusted odds ratio; SGA, small for gestational age; AGA, appropriate for gestational age; LGA, large for gestation age; NICU, neonatal intensive care unit; | | | | | | | | | | |  |
| **^a^** aOR was adjusted for maternal age, paternal age, study center, controlled ovarian stimulation protocols, embryo types. | | | | | | | | | | |  |

| **Table S6. The effect of serum AMH levels on pregnancy outcomes in ART pregnancies with frozen single embryo transfers.** | | | | | | | | | | |
| --- | --- | --- | --- | --- | --- | --- | --- | --- | --- | --- |
|  | **Low AMH (N=74)** | **Average AMH (N= 213)** | **High AMH (N=54)** |  | **Low AMH vs average AMH** | | |  | **High AMH vs average AMH** | |
|  | **n (%)** | **n (%)** | **n (%)** |  | **OR1 (95% CI)** | | **aOR1 (95% CI)^a^** |  | **OR2 (95% CI)** | **aOR2 (95% CI) ^a^** |
| **Gestational diabetes mellitus** |  |  |  |  |  | |  |  |  |  |
| No | 239 (93.7) | 843 (96.8) | 499 (96.3) |  | Reference | | Reference |  | Reference | Reference |
| Yes | 16 (6.3) | 28 (3.2) | 19 (3.7) |  | 2.02 (1.07-3.79) | | 1.18 (0.54-2.54) |  | 1.15 (0.63-2.07) | 1.07 (0.56-2.04) |
| **Hypertensive disorders in pregnancy** |  |  |  |  |  | |  |  |  |  |
| No | 234 (91.8) | 827 (94.9) | 494 (95.4) |  | Reference | | Reference |  | Reference | Reference |
| Pregnancy-induced hypertension | 13 (5.1) | 30 (3.4) | 17 (3.3) |  | 1.53 (0.79-2.98) | | 1.75 (0.80-3.80) |  | 0.95 (0.52-1.74) | 0.81 (0.41-1.62) |
| Preeclampsia | 8 (3.1) | 14 (1.6) | 7 (1.4) |  | 2.02 (0.84-4.87) | | 2.56 (0.89-7.36) |  | 0.84 (0.34-2.09) | 0.63 (0.22-1.75) |
| **Intrahepatic cholestasis of pregnancy** |  |  |  |  |  | |  |  |  |  |
| No | 253 (99.2) | 868 (99.7) | 514 (99.2) |  | Reference | | Reference |  | Reference | Reference |
| Yes | 2 (0.8) | 3 (0.3) | 4 (0.8) |  | 2.29 (0.38-13.76) | | 2.43 (0.19-31.43) |  | 2.25 (0.50-10.10) | 3.79 (0.73-19.65) |
| **Placental abnormality** |  |  |  |  |  | |  |  |  |  |
| No | 252 (98.8) | 853 (97.9) | 511 (98.6) |  | Reference | | Reference |  | Reference | Reference |
| Yes | 3 (1.2) | 18 (2.1) | 7 (1.4) |  | 0.56 (0.16-1.93) | | 0.51 (0.12-2.17) |  | 0.65 (0.27-1.56) | 0.67 (0.23-1.92) |
| **Oligohydramnios** |  |  |  |  |  | |  |  |  |  |
| No | 251 (98.4) | 857 (98.4) | 511 (98.6) |  | Reference | | Reference |  | Reference | Reference |
| Yes | 4 (1.6) | 14 (1.6) | 7 (1.4) |  | 0.98 (0.32-2.99) | | 0.62 (0.15-2.47) |  | 0.84 (0.34-2.09) | 0.90 (0.33-2.46) |
| **Premature rupture of membrane** |  |  |  |  |  | |  |  |  |  |
| No | 252 (98.8) | 834 (95.8) | 494 (95.4) |  | Reference | | Reference |  | Reference | Reference |
| Yes | 3 (1.2) | 37 (4.2) | 24 (4.6) |  | 0.27 (0.08-0.88) | | 0.09 (0.02-0.50) |  | 1.10 (0.65-1.85) | 1.10 (0.61-1.98) |
| **Mode of delivery** |  |  |  |  |  | |  |  |  |  |
| Vaginal | 63 (25.8) | 247 (29.4) | 166 (34.8) |  | Reference | | Reference |  | Reference | Reference |
| Cesarean section | 180 (73.8) | 589 (70.2) | 308 (64.6) |  | 1.20 (0.87-1.66) | | 1.12 (0.76-1.65) |  | 0.78 (0.61-0.99) | 0.75 (0.57-0.98) |
| Instrumental vaginal birth | 1 (0.4) | 3 (0.4) | 3 (0.6) |  | 1.31 (0.13-12.78) | | - |  | 1.49 (0.30-7.46) | 0.22 (0.03-1.73) |
|  |  |  |  |  |  | |  |  |  |  |
| Note: OR, odds ratio; CI, confidence interval; aOR, adjusted odds ratio; | | | | | | | | | | |
| **^a^** aOR was adjusted for maternal age, paternal age, race, study center, parity, gravidity, primary infertility, causes of infertility, controlled ovarian stimulation protocols, number of previous ART procedures, type of endometrium preparation, embryo types. | | | | | | | | | | |
| **Table S7.** **The effect of serum AMH levels on neonatal outcomes in ART pregnancies with frozen single embryo transfers.** | | | | | | | | | | |
|  | **Low AMH (N=74)** | **Average AMH (N= 213)** | **High AMH (N=54)** |  | | **Low AMH vs average AMH** | |  | **High AMH vs average AMH** | |
|  | **n (%)** | **n (%)** | **n (%)** |  | | **OR1 (95% CI)** | **aOR1 (95% CI) ^a^** |  | **OR2 (95% CI)** | **aOR2 (95% CI) ^a^** |
| **Gender** |  |  |  |  | |  |  |  |  |  |
| Male | 141 (55.3) | 482 (55.3) | 287 (55.4) |  | | Reference | Reference |  | Reference | Reference |
| Female | 114 (44.7) | 389 (44.7) | 231 (44.6) |  | | 1.00 (0.76-1.33) | 0.99 (0.71-1.39) |  | 1.00 (0.80-1.24) | 0.95 (0.74-1.21) |
| **Preterm delivery** |  |  |  |  | |  |  |  |  |  |
| No | 237 (92.9) | 815 (93.6) | 479 (92.5) |  | | Reference | Reference |  | Reference | Reference |
| Preterm | 15 (5.9) | 53 (6.1) | 35 (6.8) |  | | 1.11 (0.64-1.92) | 1.00 (0.50-1.98) |  | 1.18 (0.78-1.81) | 1.21 (0.75-1.96) |
| Very preterm | 3 (1.2) | 3 (0.3) | 4 (0.8) |  | | - | - |  | - | - |
| **Birth weight (g)** | 3400 (3150-3700) | 3400 (3100-3700) | 3450 (3150-3700) | |  | |  |  |  |  |
| <2500 | 10 (4.0) | 25 (2.9) | 23 (4.5) |  | | 1.37 (0.65-2.90) | 1.27 (0.48-3.31) |  | 1.58 (0.89-2.82) | 1.76 (0.89-3.50) |
| 2500-3999 | 221 (88.4) | 757 (87.2) | 441 (85.6) |  | | Reference | Reference |  | Reference | Reference |
| ≥4000g | 19 (7.6) | 86 (9.9) | 51 (9.9) |  | | 0.76 (0.45-1.27) | 0.97 (0.53-1.78) |  | 1.02 (0.71-1.47) | 0.86 (0.57-1.31) |
| **Weight for gestational age** |  |  |  |  | |  |  |  |  |  |
| SGA | 12 (4.8) | 27 (3.1) | 15 (2.9) |  | | 1.63 (0.81-3.27) | 1.60 (0.66-3.86) |  | 0.95 (0.50-1.81) | 0.76 (0.36-1.63) |
| AGA | 184 (73.6) | 673 (77.5) | 393 (76.3) |  | | Reference | Reference |  | Reference | Reference |
| LGA | 54 (21.6) | 168 (19.4) | 107 (20.8) |  | | 1.18 (0.83-1.66) | 0.92 (0.60-1.42) |  | 1.09 (0.83-1.43) | 0.99 (0.72-1.34) |
| **Neonatal infection** |  |  |  |  | |  |  |  |  |  |
| No | 17 (100.0) | 31 (96.9) | 28 (100.0) |  | | Reference | Reference |  | Reference | Reference |
| Yes | 0 (0.0) | 1 (3.1) | 0 (0.0) |  | | - | - |  | - | - |
| **NICU** |  |  |  |  | |  |  |  |  |  |
| No | 17 (100.0) | 30 (93.8) | 27 (96.4) |  | | Reference | Reference |  | Reference | Reference |
| Yes | 0 (0.0) | 2 (6.2) | 1 (3.6) |  | | - | 0.56 (0.05-6.48) |  | - | 0.05 (0.00-22.72) |
| **Neonatal asphyxia** |  |  |  |  | |  |  |  |  |  |
| No | 17 (89.5) | 32 (91.4) | 28 (96.6) |  | | Reference | Reference |  | Reference | Reference |
| Yes | 2 (10.5) | 3 (8.6) | 1 (3.4) |  | | 1.25 (0.19-8.25) | - |  | 0.38 (0.04-3.87) | - |
| **Neonatal jaundice** |  |  |  |  | |  |  |  |  |  |
| No | 9 (52.9) | 23 (71.9) | 22 (78.6) |  | | Reference | Reference |  | Reference | Reference |
| Yes | 8 (47.1) | 9 (28.1) | 6 (21.4) |  | | 2.27 (0.67-7.73) | 2.50 (0.37-17.04) |  | 0.70 (0.21-2.28) | 0.50 (0.09-2.65) |
| **Congenital anomaly** |  |  |  |  | |  |  |  |  |  |
| No | 239 (99.6) | 835 (99.3) | 480 (99.4) |  | | Reference | Reference |  | Reference | Reference |
| Yes | 1 (0.4) | 6 (0.7) | 3 (0.6) |  | | 0.58 (0.07-4.86) | 0.43 (0.04-4.91) |  | 0.87 (0.22-3.49) | 1.15 (0.21-6.24) |
|  |  |  |  |  | |  |  |  |  |  |
| *Note:* OR, odds ratio; CI, confidence interval; aOR, adjusted odds ratio; SGA, small for gestational age; AGA, appropriate for gestational age; LGA, large for gestation age; NICU, neonatal intensive care unit; | | | | | | | | | | |
| **^a^** aOR was adjusted for maternal age, paternal age, race, study center, parity, gravidity, primary infertility, causes of infertility, controlled ovarian stimulation protocols, number of previous ART procedures, type of endometrium preparation, embryo types. | | | | | | | | | | |
